# Design Strategies for Anodes and Interfaces Toward Practical Solid‐State Li‐Metal Batteries

**DOI:** 10.1002/advs.202302263

**Published:** 2023-08-06

**Authors:** Gabin Yoon, Sewon Kim, Ju‐Sik Kim

**Affiliations:** ^1^ Battery Material TU Samsung Advanced Institute of Technology 130, Samsung‐ro, Yeongtong‐gu Suwon‐si Gyeonggi‐do 443‐803 Republic of Korea

**Keywords:** anode interlayers, Garnet solid electrolytes, Li–metal batteries, Li metal/solid electrolyte interface

## Abstract

Solid‐state Li–metal batteries (based on solid‐state electrolytes) offer excellent safety and exhibit high potential to overcome the energy‐density limitations of current Li–ion batteries, making them suitable candidates for the rapidly developing fields of electric vehicles and energy‐storage systems. However, establishing close solid–solid contact is challenging, and Li‐dendrite formation in solid‐state electrolytes at high current densities causes fatal technical problems (due to high interfacial resistance and short‐circuit failure). The Li metal/solid electrolyte interfacial properties significantly influence the kinetics of Li–metal batteries and short‐circuit formation. This review discusses various strategies for introducing anode interlayers, from the perspective of reducing the interfacial resistance and preventing short‐circuit formation. In addition, 3D anode structural‐design strategies are discussed to alleviate the stress caused by volume changes during charging and discharging. This review highlights the importance of comprehensive anode/electrolyte interface control and anode design strategies that reduce the interfacial resistance, hinder short‐circuit formation, and facilitate stress relief for developing Li–metal batteries with commercial‐level performance.

## Graphical abstract

Solid‐state Li metal batteries have great potential in terms of safety and energy density, but stabilizing Li metal/solid electrolyte interface remains challenging. In this review, various strategies to improve interfacial properties are summarized, including surface treatments and interlayer introduction. Further, 3D anode design strategies which can relieve the stress at the interface are reviewed.

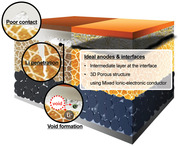

## 1 Introduction

Solid‐state battery technology has gained prominence due to the increasing demand for safe rechargeable batteries with high energy density. Compared with conventional Li–ion batteries that use liquid electrolytes, solid‐state batteries offer higher safety and significantly higher volumetric energy densities.^[^ ^1^, ^2^ ^]^ This is because solid electrolytes are non‐flammable and enable the use of Li metal as the anode. Further, solid‐state batteries, with a wide operating‐temperature range that facilitates an increase in the energy density of battery modules and packs, can simplify cooling systems.

However, constructing commercial high‐performance solid‐state batteries requires the resolution of several issues related to Li metal–solid electrolyte interfaces.^[^ ^3^, ^4^, ^5^, ^6^ ^]^ First, chemical impurities and physical defects on the solid–electrolyte surface make it challenging to establish intimate contact between the solid electrolyte and Li metal. Insulating phases, such as Li_2_CO_3_ and LiOH, which form on the solid–electrolyte surface due to reactions with water and/or CO_2_ in ambient air, act as inactive sites and increase the initial charge‐transfer resistance for Li plating/stripping.^[^ ^7^, ^8^ ^]^ Further, the slow diffusion of neutral Li atoms causes void formation or the delamination of the Li–metal anode at the solid electrolyte interface during discharging. This phenomenon is exacerbated under high‐current conditions, where the rate of Li‐atom consumption owing to Li stripping exceeds the kinetics of Li‐atom diffusion in the Li metal. These voids persist in subsequent charging cycles and act as inactive sites for Li plating/stripping, leading to an elevated interfacial resistance and non‐uniform current distribution.^[^ ^6^, ^9^, ^10^ ^]^

In addition, Li plating and stripping during charging and discharging causes anode expansion and contraction. Volume expansion during the charging process can cause mechanical fatigue in the anode and solid electrolyte because of the high hydrostatic pressure generated by the deposited Li, leading to stress‐induced damage at the solid electrolyte–anode interface. This accumulated‐strain damage is a major cause of crack generation and propagation in solid electrolytes and delamination at the solid electrolyte/electrode interface.^[^ ^11^, ^12^, ^13^ ^]^ These mechanical‐stress issues caused by Li expansion and contraction are expected to be more critical in multi‐stack cells with multiple layers of solid electrolytes.

Issues arising at the solid electrolyte/Li metal anode interface impede the stable maintenance of the interface and increase the initial interfacial resistance. Moreover, they cause a local current concentration, which generates a nonuniform current distribution at the interface. These issues, such as Li‐dendrite formation, are among the biggest technical challenges in solid‐state‐electrolyte research, and resolving them is vital for the commercialization of Li–metal batteries based on solid‐state electrolytes. Effective interface‐control and anode‐design strategies are required to suppress void formation and maintain strong interfacial adhesion between the solid electrolyte and Li metal, while alleviating the stress induced by volume changes in the Li‐metal anode.

**Figure 1:**
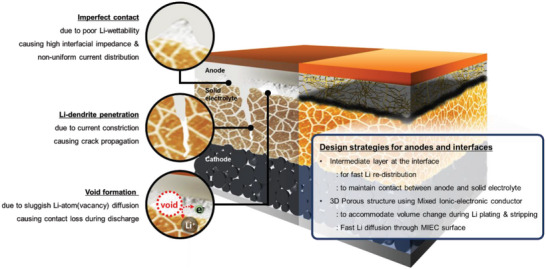
Issues related to the anodes and Li metal–solid electrolyte interfaces. Design strategies to control the Li metal–solid electrolyte interface are summarized.

This review summarizes several strategies to control the Li metal/solid electrolyte interface (such as interlayer introduction and the surface treatment of solid‐state electrolytes), which can help overcome the technical challenges associated with such interfaces in Li‐metal all‐solid‐state batteries (**Figure** **1**). In addition, 3D anode‐design strategies for stress relief at the Li metal/solid electrolyte interface caused by changes in the Li‐anode volume during charging and discharging are discussed, focusing on garnet‐based solid oxide electrolytes that are chemically and electrochemically stable with Li metal, and exhibit acceptable interfacial stability. However, the problems at the Li metal/solid electrolyte interface are not unique to Li‐metal batteries with solid oxide electrolytes. In fact, the mechanism of Li dendrite formation at the anode/electrolyte interface and the Li plating/stripping reactions in lithium metal anodes are fundamentally the same in other inorganic solid electrolytes, such as sulfide‐based electrolytes. The comprehensive interfacial control and anode design strategies presented in this paper could therefore be considered to be universally applicable to other lithium metal batteries based on ceramic solid electrolytes.

## 2 Li Metal/Solid Electrolyte Interface‐Control Strategies

### 2.1 Strategies to Reduce the Li Metal/Solid Electrolyte Interfacial Resistance

Point‐contact formation makes it challenging to establish conformal contact between rigid inorganic solid electrolytes and Li‐metal anodes. The application of high external pressures (>250 MPa) and utilizing molten Li metal to attach the Li‐metal anode to the solid electrolyte have been used to ensure intimate contact (without point contact).^[^ ^14^, ^15^ ^]^ However, surface impurities inevitably form on the solid electrolyte, causing poor physical contact between the Li metal and solid electrolyte, resulting in high interfacial resistance.^[^ ^7^, ^16^ ^]^ For example, garnet‐structured oxide electrolytes (Li_7_La_3_Zr_2_O_12_, denoted LLZO) exhibit poor Li wettability because of the formation of lithiophobic phases (such as LiOH and Li_2_CO_3_) on their surface due to reactions with water and CO_2_ in ambient air.^[^ ^16^ ^]^

Conformal contact with Li metal has been realized by removing the impurities responsible for poor Li metal wettability, such as Li_2_CO_3_ and LiOH, from the solid–electrolyte surface. Initial attempts involved the introduction of heat treatment and physical polishing as effective methods for eliminating the impurities and defects on the surface of a solid electrolyte.^[^ ^17^, ^18^, ^19^ ^]^ Sakamoto et al. used mechanical polishing with sandpaper, followed by heat treatment (at 500 °C) in an Ar atmosphere, to largely eliminate impurities (such as hydroxides and carbonates) from the surfaces of Al‐doped LLZTO (Ta‐doped LLZO) solid electrolytes, thereby lowering the interfacial impedance to 2 Ω cm^2^.^[^ ^17^ ^]^ Goodenough et al. removed Li_2_CO_3_ and protons from the electrolyte surface using heat treatment with carbon at 700 °C. They subsequently confirmed that the interfacial resistance had been lowered by the removal of the impurities by analyzing the impedance of symmetrical Li metal cells based on heat‐treated LLZTO as the solid electrolyte.^[^ ^18^ ^]^ Laser annealing was also shown to be suitable for lowering the interfacial resistance by removing the impurities from the surface of LLZTO solid electrolytes.^[^ ^20^ ^]^ Importantly, laser treatment of the surface not only removed impurities but also formed the Li_2_O_2_ and amorphous garnet phases on the surface, which lowered the electronic conductivity and inhibited Li‐dendrite formation.

**Figure 2:**
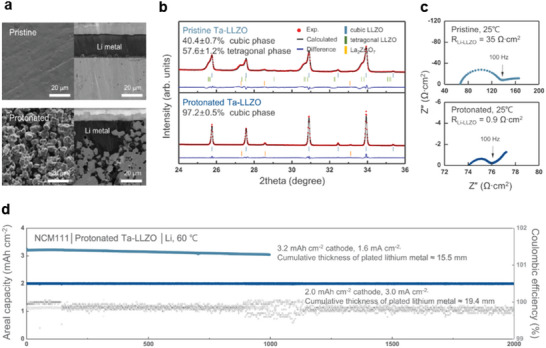
a) Cross‐sectional SEM images of a polished and acid‐treated Ta–LLZO (LLZTO) solid electrolyte placed in direct contact with Li metal. b) XRD patterns of the LLZTO before and after treatment with acid. c) Electrochemical impedance spectra of Li/LLZTO/Li symmetric cells before and after exposing the surface to HCl (1 m). d) Long‐term cycling performance of a quasi‐solid‐state battery cell (Li|acid‐treated LLZTO|NCM111‐ionic liquid) at cathode capacities of 3.2 and 2.0 mAh cm^−2^. Reproduced with permission.^[^ ^15^ ^]^ Copyright 2022, Springer Nature.

Several studies have attempted to improve the Li‐wetting characteristics of solid electrolytes by using chemical etching to remove impurities from their surfaces.^[^ ^15^, ^21^, ^22^, ^23^ ^]^ The Li_2_CO_3_ that is inevitably present on the surface of garnet solid‐oxide electrolytes can be effectively removed by exposure to strong acids, such as H_3_PO_4_ and HCl. Solutions of these acids undergo reduction–decomposition reactions with the impurity. The use of acid to remove impurities increases the surface area of the solid electrolyte as a result of surface etching, which is expected to increase the electrochemically active area in contact with the Li metal, as shown in **Figure** **2**a.^[^ ^15^ ^]^ Moreover, acid treatment has the effect of partially protonating garnet and decreasing its surface tetragonal phases; this can increase the mechanical strength of the solid‐electrolyte film, owing to a decrease in the lattice strain (Figure 2b). According to this study,^[^ ^15^ ^]^ the room‐temperature interfacial resistance of LLZTO pellets decreased to 0.9 Ω cm^2^ after surface treatment with HCl (1 m), without an anode interlayer, as indicated by the impedance spectra of symmetric Li cells (Figure 2c). Moreover, these cells exhibited excellent long‐term cycling stability of more than 1000 cycles at 0.5 C and 60 °C in a quasi‐solid‐state battery cell (Li|LLZTO|NCM111 with ionic liquid) with 3.2 mAh cm^−2^ capacity (Figure 2d). Collectively, these results indicate that the lithiophilic properties of solid electrolytes can be improved by removing surface impurities. This obviates the need for the introduction of an anode interlayer, thereby facilitating conformal contact between the solid electrolyte and Li‐metal anode.

**Figure 3:**
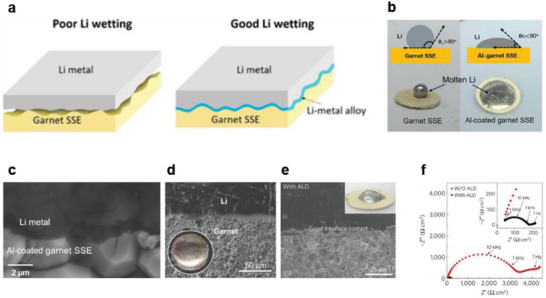
a) Schematic representations of the Li wettability of solid‐state electrolytes (SSEs) without and with a metal interlayer. Reproduced with permission.^[^ ^14^ ^]^ Copyright 2017, American Association for the Advancement of Science. b) Digital images and schematic illustrations showing the wetting behavior and contact angles of a pristine SSE and Al‐coated SSE. Reproduced with permission.^[^ ^14^ ^]^ Copyright 2017, American Association for the Advancement of Science. c) Cross‐sectional SEM image of the interface between an Al‐coated garnet SSE and Li metal. Reproduced with permission.^[^ ^14^ ^]^ Copyright 2017, American Association for the Advancement of Science. Cross‐sectional SEM images of the Li metal and SSE interface with d) ZnO. Reproduced with permission.^[^ ^24^ ^]^ Copyright 2016, American Chemical Society; and e) Al_2_O_3_. Reproduced with permission.^[^ ^25^ ^]^ Copyright 2016, Springer Nature. Inset: digital images showing the Li‐wetting behavior of the SSEs. f) Nyquist plots of ac‐impedance spectra recorded using Li symmetric cells with garnet SSEs, without and with an Al_2_O_3_ coating layer; inset: enlarged impedance spectra of the symmetric cells. Reproduced with permission.^[^ ^25^ ^]^ Copyright 2016, Springer Nature.

Several studies have attempted to establish an intimate contact interface between solid electrolytes and Li metal by introducing various lithiophilic interfacial layers or manipulating the solid electrolyte surface, as shown in **Figure** **3**a. Metal interlayers have been introduced into the Li metal/solid electrolyte interface to improve the Li metal interfacial bonding.^[^ ^14^, ^26^, ^27^, ^28^, ^29^, ^30^, ^31^, ^32^ ^]^ Further, multiple studies have attempted to improve the Li wettability of solid electrolytes by coating their surface with metalloid or metal components such as Ag,^[^ ^26^ ^]^ Si,^[^ ^27^ ^]^ Au,^[^ ^28^, ^29^ ^]^ Al,^[^ ^14^ ^]^ Ge,^[^ ^30^ ^]^ Mg,^[^ ^31^ ^]^ and Zn;^[^ ^32^ ^]^ these materials can form alloys with Li and make compact contact with garnet solid electrolytes. In the case of an Ag interlayer, the electrochemical reaction kinetics can be expected to improve because of the higher Li diffusivity of the LiAg alloy (10^−8^ cm^2^s^−1^) compared with that of pure Li metal (5.7 × 10^−11^ cm^2^s^−1^). The Ag interlayer additionally serves to enhance the Li wettability of solid electrolytes.^[^ ^33^ ^]^ In these studies, various deposition methods (plasma‐enhanced chemical vapor deposition, electron beam evaporation, and sputtering) are used to deposit a thin interfacial metal layer (a thickness of less than 100 nm) on the solid electrolyte to improve its Li wettability. As shown in Figure 3b, the contact angle of molten Li on a garnet solid‐state electrolyte (SSE) at 200 °C drastically decreases on coating the SSE with Al.^[^ ^14^ ^]^ The cross‐sectional SEM images in Figure 3c indicate that the interfacial metal layer, when placed in direct contact with Li metal, induces the formation of an intimate and uniform Li metal/SSE interface through an in situ alloy reaction. Density functional theory (DFT) calculations on the interfacial reaction energy between Si and Nb interfacial metal layers with Ca‐doped LLZO electrolytes confirm that the introduction of lithiophilic metal layers enhances the adhesion of SSEs with Li metal.^[^ ^14^ ^]^

As an alternative method to improve the contact properties of SSEs through alloying reactions, several studies have attempted to improve Li wettability through the conversion reactions between inorganic materials (oxides, nitrides, sulfides, and fluorides) and lithium metal.^[^ ^24^, ^25^, ^34^, ^35^, ^36^ ^]^ As shown in the SEM images in Figure 3d,e, ZnO‐ and Al_2_O_3_‐coated SSEs form conformal contacts with Li metal (without any voids) due to good Li wetting, similar to SSEs with an Al–metal interlayer (Figure 3c). According to Wachsman et al., the Li‐wetting property of solid electrolytes can be improved by the deposition of ultrathin Al_2_O_3_ on the electrolyte surface, followed by contact with molten Li; the interfacial resistance is greatly reduced to 1 ohm cm^2^, as shown in the impedance spectra in Figure 3f.^[^ ^25^ ^]^ First‐principles calculations attribute the enhanced contact property of the oxide‐coated SSE to the higher binding energy of Li*_x_* Al_2_O_3+_ *_x_*_/2_ (*x* = 0.4 to 1.4; formed by the conversion reaction between Li and Al_2_O_3_) compared to that of Li_2_CO_3_. Recently, melting‐quenching methods have been used for coating solid electrolytes with metal oxides.^[^ ^36^ ^]^ Cui et al. uniformly coated an LLZTO solid electrolyte with a thin Cu*_z_* Sn*_y_* O*_x_* (6/5 < *x*/*y* < 3) interlayer (with strong adhesion to the solid electrolyte) by attaching a molten Cu–Sn alloy to LLZTO, followed by cooling. Despite low cathode‐loading (5 mg cm^−2^), all‐solid‐state Li‐NMC(LiNi_0.8_Mn_0.1_Co_0.1_O_2_) cells with the Cu*_z_* Sn*_y_* O*_x_*‐coated LLZTO electrolyte exhibited stable cycling performance over 1000 cycles without an internal short circuit at room temperature.^[^ ^36^ ^]^ Finally, several approaches involving the introduction of oxide layers by utilizing conversion reactions between lithiophobic layers (LiOH, Li_2_CO_3_) on the surface have been reported.^[^ ^37^, ^38^ ^]^ Bi et al. effectively decreased the interfacial resistance by replacing the surface contaminant Li_2_CO_3_ with a Li_3_PO_4_ layer with the aid of a conversion reaction driven by the salt NH_4_H_2_PO_4_.^[^ ^37^ ^]^ Similarly, Cai et al. deposited lithiophilic metal oxides such as PbO, ZnO, and Co_3_O_4_ by exploiting the conversion reaction between LiOH and aqueous solutions of the metal salt to successfully lower the interfacial resistance.^[^ ^38^ ^]^

In addition to oxides, nitrides, sulfides, and fluorides have been investigated as interlayers.^[^ ^34^, ^35^, ^39^, ^40^ ^]^ Baniya et al. have deposited Si_3_N_4_ on a garnet solid‐electrolyte and attached it to Li metal to form a Li‐ion conducting Li_8_SiN_4_ layer via a conversion reaction,^[^ ^35^ ^]^ whereas Fu et al. have introduced MoS_2_ to induce the formation of an Mo + Li_2_S layer, thereby reducing the interfacial resistance.^[^ ^34^ ^]^ In systems containing MoS_2_, the inorganic interlayer (Mo + Li_2_S) formed by conversion reactions improves the Li wettability of the electrolyte and facilitates current redistribution through the anode interlayer by blocking electron transport at the anode–electrolyte interface.^[^ ^34^ ^]^ Cai et al. introduced a porous composite layer composed of LiF and Li*_x_* BO*_y_* on the surface of the LLZTO electrolyte by using drop coating,^[^ ^40^ ^]^ and Duan et al. coated LLZTO with a layer of LiF by using NH_4_F treatment.^[^ ^39^ ^]^ Li ion‐conductive inorganic interlayers formed at the anode/solid electrolyte interface by such conversion reactions are expected to exhibit higher stability than metal interlayers, even after cycling, due to the absence of metal diffusion from the inorganic layer toward the Li metal. Thus, in systems with inorganic interlayers coated on the SSE, the Li metal anode is mainly composed of pure Li metal rather than a Li metal solid solution or alloy.

Intermediate layers have been used to enhance the contact properties of SSEs with Li metal and reduce their interfacial resistance. Interfacial coating layers increase the contact area between the solid–electrolyte interface and Li‐metal anode, maximizing the effective area for Li plating/stripping reactions and reducing the interfacial resistance significantly. Although the formation of a single thin‐layer effectively reduces the initial interfacial resistance by improving the Li wettability of the solid electrolyte, it is challenging to control the short‐circuit formation caused by Li permeation through the solid electrolyte. First, the physical bonding strength between the solid electrolyte and anode interlayer gradually degrades with long‐term repetitive charging/discharging owing to volume changes in the anode.^[^ ^41^, ^42^ ^]^ During the charging and discharging of an Li‐ion battery, Li plating/stripping and anode‐interlayer alloying/de‐alloying or conversion/deconversion reactions cause Li‐metal volume changes. This causes void formation and delamination at the anode/electrolyte interface, making it difficult to maintain intimate contact between the Li metal and solid electrolyte. In addition, when a strong electric field is applied to a solid electrolyte, minor carrier electrons flow through the grain boundaries or surface defects in a relatively high‐energy state owing to dielectric breakdown, resulting in the formation of Li dendrites.^[^ ^43^, ^44^ ^]^ A single interlayer composed of a metal or alloy with electron conductivity cannot prevent local electron flow on the solid–electrolyte surface. Even for a single inorganic layer, the Li‐dendrite resistance may be lower due to the formation of electron‐conducting metal compounds during the conversion process.

**Figure 4:**
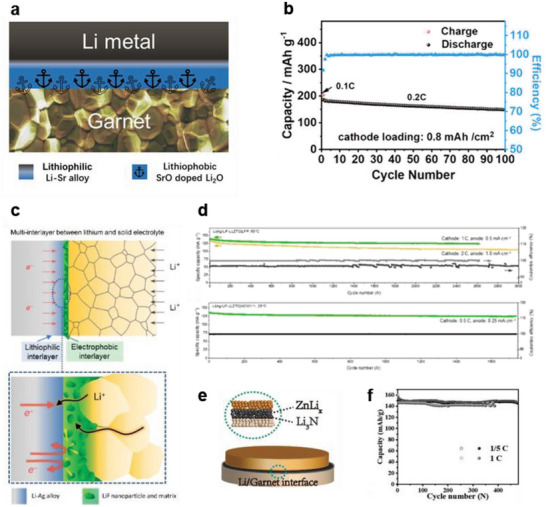
a) Schematic illustration of an Li–Sr/SrO bilayer on a garnet SSE. Reproduced with permission.^[^ ^45^ ^]^ Copyright 2016, American Chemical Society. b) Cycling data for an Li‐Sr|SrO/LLZTO|NCM811 cell at 0.2 C with a cathode capacity of 0.8 mAh cm^−2^. Reproduced with permission.^[^ ^45^ ^]^ Copyright 2016, American Chemical Society. c) Schematic diagrams of the Li metal/garnet SSE interface with lithiophilic Li–Ag alloy and electron‐blocking LiF interlayers. Reproduced with permission.^[^ ^46^ ^]^ Copyright 2022, American Association for the Advancement of Science. d) Cycle performances of Li–Ag|LiF‐coated LLZTO|LFP and Li–Ag|LiF‐coated|NCM111 cells at 60 °C and 25 °C, respectively. Reproduced with permission.^[^ ^46^ ^]^ Copyright 2022, American Association for the Advancement of Science. e) Schematic diagram of the Li metal and garnet SSE interface. Reproduced with permission.^[^ ^47^ ^]^ Copyright 2020, Wiley‐VCH. f) Cycle performances of the Li|Li‐Zn/Li_3_N/LLZTO|LFP at rates of 0.2 and 0.5 C. Reproduced with permission.^[^ ^47^ ^]^ Copyright 2020, Wiley‐VCH.

To overcome these limitations, recent studies have attempted to increase the limiting current density by constructing a dual interlayer consisting of an Li alloy layer (to accommodate volume changes) and inorganic layer (to block electrons).^[^ ^45^, ^46^ ^]^ As shown in **Figure** **4**a, He et al. have fabricated a bifunctional layer composed of a lithiophilic Li–Sr alloy and lithiophobic SrO on the surface of an LLZTO solid electrolyte by placing the Li–Sr alloy and solid electrolyte in direct contact with each other at 300 °C. When the molten Li–Sr alloy is attached to the solid electrolyte, an interfacial reaction layer (SrO) is formed at the Li–Sr alloy/LLZTO interface.^[^ ^45^ ^]^ As SrO is lithiophobic and extremely compatible with solid oxide electrolytes, it pushes the plated Li toward the lithiophilic Li–Sr layer during the charging process, thereby preventing local Li deposition on the LLZTO surface and suppressing Li‐dendrite formation. Thus, an Li‐Sr/SrO/LLZTO/NCM811 cell with an anode bilayer shows a stable cycle performance of 100 cycles at 0.16 mA cm^−2^ and room temperature (Figure 4b).

Kang et al. have coated a bilayer composed of LiF and Ag onto a solid electrolyte using a vacuum thermal evaporator, followed by attachment to molten Li metal, as shown in Figure 4c. In the bilayer, the LiF layer consists of ≈10 nm nanoparticles dispersed in an amorphous matrix; it plays a role in inhibiting electron flow.^[^ ^46^ ^]^ The electron‐blocking bilayer prevents electron leakage at the solid–electrolyte interface, enabling Li‐metal cells with LLZTO electrolytes to exhibit long‐term cycling without short‐circuiting, as shown in Figure 4. Li‐Ag|LiF/LLZTO|LFP and Li‐Ag|LiF/LLZTO|NCM111 cells exhibit stable cycling (3000 cycles at 60 °C and 1600 cycles at 25 °C) without short‐circuit failure.^[^ ^46^ ^]^

Using a similar approach, research has been conducted on functional bilayers such as Li–Zn/Li_3_N^[^ ^47^ ^]^ and Li–Sn/LiF.^[^ ^48^ ^]^ These double layers have been fabricated by a wet coating method instead of the complicated and expensive deposition method. For instance, to form a Li–Zn/Li_3_N anode, as shown in Figure 4e, Zn(NO_3_)_2_ and SnF_2_ solutions have been coated onto LLZTO solid electrolytes by drop coating, followed by a chemical conversion reaction with molten Li metal. These robust Li binary inorganic layers, in direct contact with the solid electrolyte, exhibit low electron conductivity and small volume changes during Li plating/stripping, preventing electron flow at the solid electrolyte interface and maintaining good adhesion with the solid electrolyte. With these double interlayers, the room‐temperature critical current densities for symmetric cells consisting of Li–Sn/LiF/LLZTO and Li–Zn/Li_3_N/LLZTO are improved to high values of up to 2.4 and 2.0 mA cm^−2^, respectively. As shown in Figure 4f, the full cell Li|Li‐Zn/Li_3_N/LLZTO|LFP exhibits stable cycling performance at 0.2 and 1 C.

**Figure 5:**
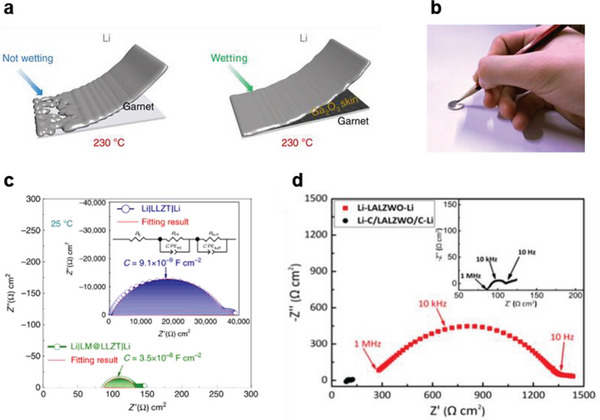
a) Schematic illustration of the wetting behavior of molten Li on SSE surfaces without and with Ga liquid–metal coating. The liquid metal is painted on the garnet SSE at 230 °C. Reproduced with permission.^[^ ^49^ ^]^ Copyright 2020, Springer Nature. b) A digital image of the preparation of a graphite interlayer by pencil drawing. Reproduced with permission.^[^ ^50^ ^]^ Copyright 2018, American Chemical Society. c) Electrochemical impedance spectra of Li symmetric cells without and with a liquid–metal interlayer at 25 °C. Reproduced with permission.^[^ ^49^ ^]^ Copyright 2020, Springer Nature. d) Electrochemical impedance spectra of Li symmetric cells without and with a graphite interlayer at 25 °C. Reproduced with permission.^[^ ^50^ ^]^ Copyright 2018, American Chemical Society.

In addition to thin anode interlayers composed of a metal alloy or metal oxide, liquid metal and graphite have also been investigated as lithiophilic‐induced interlayers for good contact between solid electrolytes and Li metal.^[^ ^49^, ^50^ ^]^ These interlayers can be easily fabricated by simple coating processes such as slurry painting and pencil drawing, as shown in **Figure** **5**a,b. Meng et al. have coated a liquid metal (such as Ga, with an excellent affinity for LLZTO and Li_2_CO_3_) on LLZTO solid electrolytes by brush painting, reducing the interfacial resistance with Li metal to 5 Ω cm^2^ without Li_2_CO_3_ removal from the solid–electrolyte surface (Figure 5c).^[^ ^49^ ^]^ Shao et al. have coated high‐ductility graphite on LLZTO using the pencil‐drawing method to reduce the initial impedance of the solid electrolyte from 1350 to 105 Ω cm^2^ (Figure 5d).^[^ ^50^ ^]^

Lithium‐ion conducting polymers have also been used as a lithiophilic buffer layer for solid oxide electrolytes.^[^ ^51^, ^52^, ^53^, ^54^, ^55^, ^56^ ^]^ Their inherent flexibility and elasticity enable them to form conformal interfaces between the lithium metal and rigid solid oxide electrolytes to enhance the wetting with lithium metal. The application of polymer coatings based on poly(ethylene oxide) or poly(propylene carbonate) to NASICON‐type solid electrolytes decreases the interfacial resistance to the level of ≈99–150 Ω cm^2^.^[^ ^51^, ^52^ ^]^ The polymer interlayers also play a role in improving the interfacial stability of solid electrolytes such as Li_1.5_Al_0.5_Ge_1.5_(PO_4_)_3_ (LAGP) or Li_1.5_Al_0.5_Ti_1.5_(PO_4_)_3_ (LATP), which are electrochemically unstable in contact with Li metal, by preventing these electrolytes from undergoing decomposition with the lithium metal. Further, the polymer layers can induce a uniform Li^+^ flux at the interface between the lithium metal anode and solid electrolyte; thus, suppressing dendrite formation.^[^ ^53^ ^]^ For instance, Zhou et al. reported that a layer of cross‐linked poly(ethylene glycol) increased the cycling performance of a Li/LLZO/LiFePO_4_ cell by preventing selective lithium deposition at the grain boundaries of LLZO.^[^ ^55^ ^]^ Compared to metal and metal oxide‐based interlayers; however, polymers might have a certain disadvantage in terms of Li diffusion kinetics and long‐term stability because of their relatively low ionic conductivity and low lithium transference number.^[^ ^57^, ^58^, ^59^ ^]^

The aforementioned strategies (involving the enhancement of the interfacial Li‐wetting properties) for generating conformal contact between solid electrolytes and Li‐metal anodes effectively reduce the initial interfacial resistance. This increases the effective area for Li plating/stripping, which improves the current–density uniformity and the effective current density at the anode. Therefore, strategies generating an intimate interface between solid electrolytes and Li metal reduce the charge‐transfer resistance at the anode/solid electrolyte interface and improve the critical current density to a certain extent.

However, considering the current density of commercial batteries in IT devices and electric vehicles, the critical current density requires further improvement. In particular, at low temperatures, a low initial interfacial resistance is not an absolute indicator of a high critical current density. Several cells with anode interlayers and surface‐treated solid electrolytes show single‐digit interfacial resistance values; however, most of them do not exhibit adequately high levels of current density (≥3 mA cm^−2^) and the long lifespan required for commercial applications. Thus, Li permeation through solid electrolytes cannot be completely eliminated by the formation of a Li metal/solid electrolyte conformal interface. During repeated charging and discharging cycles, physical defects, such as voids and stress‐induced cracks, can occur in the Li metal/solid electrolyte interface, owing to volume changes in the Li‐metal anode. These interface defects can trigger a nonuniform current distribution at the interface, leading to Li‐dendrite formation in the solid electrolytes. This Li‐dendrite formation is exacerbated at low temperatures (where the Li‐atom mobility is low), limiting the operating‐temperature range of Li‐metal batteries with solid electrolytes. Therefore, in addition to the formation of a conformal interface, interface control and anode‐structure design strategies are required to prevent short‐circuit failure.

### 2.2 Strategies to Maintain a Conformal Interface During Prolonged Cycling

As mentioned previously, introducing an artificial interlayer between the LLZO electrolyte and Li‐metal anode can effectively reduce the interfacial resistance by forming a conformal interface. However, reducing the initial interfacial resistance or generating intimate contact at the interface alone does not ensure a practicable battery‐cell performance because the interface changes dynamically during battery operation, continuously altering the physical contact between the solid electrolyte and Li metal, depending on the morphological change of the Li‐metal anode during repetitive Li plating and stripping.^[^ ^6^, ^60^, ^61^, ^62^, ^63^, ^64^, ^65^ ^]^ Therefore, to ensure the prolonged stability of the Li metal/solid electrolyte interface, it is crucial to understand the interface dynamics and continuously maintain intimate contact during battery operation (not only at the beginning).

**Figure 6:**
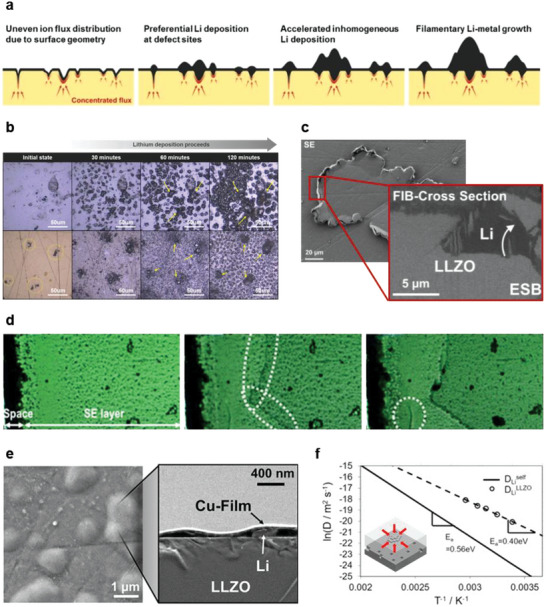
a) Schematic illustration of an uneven Li‐ion flux induced by surface‐morphology fluctuations and the corresponding inhomogeneous Li deposition. Reproduced with permission.^[^ ^66^ ^]^ Copyright 2020, Wiley‐VCH. b) In‐operando optical microscopy images of Li deposition on: (top) a precoated thick (≈1 µm) Li layer and (bottom) a polished LLZO surface with a sputtered thin (≈5 nm) gold layer during the galvanostatic deposition of Li at a constant current density (0.1 mA cm^−2^). Reproduced with permission.^[^ ^66^ ^]^ Copyright 2020, Wiley‐VCH. c) SEM image (left) of the dendrite‐like growth of Li metal underneath a copper thin‐film, with an FIB‐SEM cross‐section (right image) image of the red‐box region in the SEM image. The cross‐section image indicates an intragranular deposition of Li, which cracks the solid electrolyte. Reproduced with permission.^[^ ^60^ ^]^ Copyright 2019, Elsevier. d) Optical microscope images of an Li_2_S–P_2_S_5_ solid‐electrolyte layer and the solid electrolyte/stainless steel‐foil interface, before (left) and after Li deposition at 2 mA cm^−2^ (middle) and 100 mA cm^−2^ (right). On continuously applying a very high current density of 20 mA cm^−2^ after the short circuit, the existing cracks become larger. The formation of cracks may be caused by stress due to the dendritic growth of Li. Reproduced with permission.^[^ ^68^ ^]^ Copyright 2013, The Royal Society of Chemistry. e) SEM images of the (left) surface and (right) cross section after Li‐metal plating using a copper current collector; a mechanically broken cross section confirms spatially inhomogeneous Li nucleation underneath the copper current‐collector film. Reproduced with permission.^[^ ^60^ ^]^ Copyright 2019, Elsevier. f) The diffusivity of Li in LLZO (calculated from the Nernst–Einstein equation) compared to the self‐diffusivity of Li. Reproduced with permission.^[^ ^69^ ^]^ Copyright 2019, Elsevier.

Several groups have reported that non‐uniform Li plating due to the inhomogeneity or defects of solid electrolyte surfaces can deteriorate the interfacial contact, as shown in **Figure** **6**a.^[^ ^60^, ^66^, ^67^, ^68^ ^]^ Kim et al. have reported non‐uniform Li‐metal plating on garnet‐type electrolytes using an operando optical‐microscope system. As shown in Figure 6b, Li metal preferentially grows at the defect sites, such as pinholes, scratches, and cracks.^[^ ^60^, ^66^ ^]^ The non‐uniform plating of Li metal increases the susceptibility of Li metal penetration through the solid electrolyte (Figure 6c), inducing crack formation (Figure 6d), thereby causing battery failure. Moreover, localized Li metal growth can tear or lift current collectors, causing a contact‐loss and increase in interfacial resistance, as shown in Figure 6e.^[^ ^66^, ^68^ ^]^ Thus, interfacial instability and cell failure can be attributed to a non‐uniform current distribution on the solid–electrolyte surface and slow Li diffusion in Li metal. Wang et al. have compared the diffusion coefficient of Li^+^ in LLZO to the self‐diffusion coefficient of Li^0^ in Li metal, confirming Li^0^‐transport in Li metal to be significantly slower than Li^+^‐transport in LLZO, as shown in Figure 6f.^[^ ^69^ ^]^ Thus, Li atoms accumulate unevenly at hot spots where Li^+^ ions traverse the interface faster than the rate at which Li atoms diffuse out of the Li metal. This Li accumulation facilitates nonuniform Li plating and acts as the seed for Li metal penetration. These observations indicate that the Li metal/solid electrolyte interface undergoes facile transformation during battery operation.

**Figure 7:**
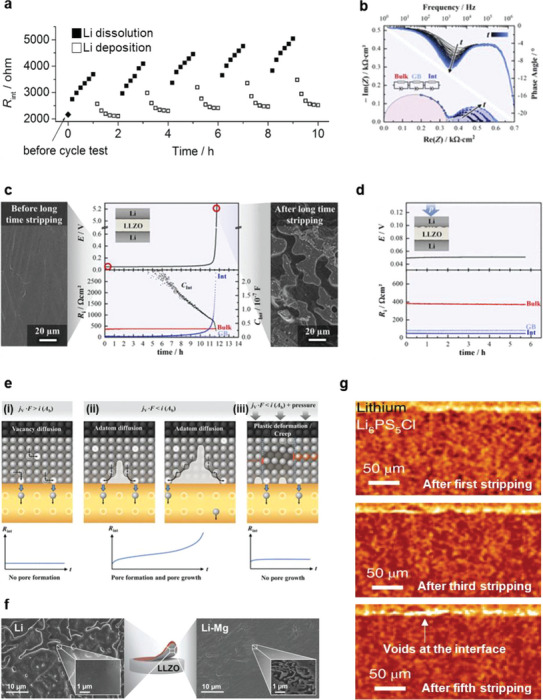
a) Interfacial resistance at the Li (W.E.)/LLZ interface as a function of time, on dissolving and depositing a Li‐metal layer (with a thickness of 250 nm) at 50 µA cm^−2^ in a three‐electrode cell. Reproduced with permission.^[^ ^62^ ^]^ Copyright 2017, Elsevier. b) Results of a Li‐stripping GEIS measurement of a Li|LLZO|Li_bc_ cell with an apparent current density of 100 µA cm^−2^. Evolution of the impedance with time is shown in the Nyquist representation, and for all cycles using a phase angle versus frequency plot during the dissolution process. Reproduced with permission.^[^ ^61^ ^]^ Copyright 2019, American Chemical Society. c) Morphology of the Li‐metal anode side facing the solid electrolyte (left) before assembling the cell, and (right), after prolonged stripping at an anodic load of 100 µA cm^−2^, with (middle) the potential profile and extracted impedance contributions. Reproduced with permission.^[^ ^61^ ^]^ Copyright 2019, American Chemical Society. d) The voltage profile and area‐specific resistances under an external pressure of ≈35 MPa, indicating a complete elimination of pore formation, which causes contact loss. Reproduced with permission.^[^ ^61^ ^]^ Copyright 2019, American Chemical Society. e) Schematic representation of the interfacial‐morphology changes for different mechanisms of Li stripping: e‐i) Stable interface with a local current density that does not exceed the vacancy‐diffusion limit in the metal. e‐ii) Contact loss due to a higher local current density (exceeding the Li vacancy diffusion rate). e‐iii) Restricted contact‐loss owing to the external pressure, which induces the plastic deformation of lithium, causing pore annihilation. Reproduced with permission.^[^ ^61^ ^]^ Copyright 2019, American Chemical Society. f) SEM images of Li metal and Li–Mg alloy electrodes after stripping experiments at *i* = 100 µA cm^−2^. Reproduced with permission.^[^ ^70^ ^]^ Copyright 2019, Wiley‐VCH. g) In situ X‐ray computed tomography images of the Li metal/Li_6_PS_5_Cl interface of a cycled Li metal/Li_6_PS_5_Cl/Li metal cell. Reproduced with permission.^[^ ^65^ ^]^ Copyright 2019, Springer Nature.

Notably, interfacial contact‐loss or deterioration is significantly pronounced/aggravated during Li stripping.^[^ ^6^, ^60^, ^61^, ^62^, ^65^, ^70^ ^]^ According to Koshikawa et al., the interfacial resistance between the Li‐metal anode and LLZO electrolyte notably increases during Li stripping.^[^ ^62^ ^]^ EIS analysis using a three‐electrode system (to investigate the dynamic changes in the Li/LLZO interface resistance during repetitive cycling of electrochemical Li plating/stripping) indicates that the interfacial resistance increases more prominently during the Li‐stripping process than during the plating process (**Figure** **7**a). This can be attributed to the formation of voids at the interface during Li stripping, which causes a contact‐loss at the interface, as confirmed by operando galvanostatic electrochemical impedance spectroscopy (GEIS), which can detect real‐time electrode impedance changes under the operating conditions.^[^ ^61^, ^70^ ^]^ Krauskopf et al. have used GEIS measurements to examine the interface kinetics of a Li‐metal anode in contact with an LLZO electrolyte under a current load. As shown in Figure 7b, the interfacial resistance increases on prolonged Li stripping, causing a corresponding increase in the cell overpotential, as shown in Figure 7c. Interestingly, the interfacial capacity decreases during dissolution, indicating a reduction in the effective contact area between the solid electrolyte and Li‐metal anode. Li metal forms a rough surface with voids after prolonged stripping, whereas pristine Li metal shows a smooth morphology without voids or craters. However, a Li/LLZO/Li symmetric cell (under an external pressure of 35 MPa) does not show significant increments in the cell overpotential and interfacial resistance during Li stripping (Figure 7d). Thus, vacancy‐diffusion in Li metal significantly influences its contact at the interface. As shown in Figure 7e, when the local current‐density during Li stripping exceeds the vacancy‐diffusion limit in Li metal, the vacancies are supersaturated and accumulate at the interface, forming pores and causing a contact loss and morphological instability. However, because external pressure induces plastic and creep deformation in Li metal, leading to pore annihilation, it continues to maintain contact with the solid electrolyte. Moreover, Li alloy electrodes exhibit high morphological stability by mitigating the depletion of active sites at the interface.^[^ ^70^ ^]^ As shown in Figure 7f, Li–Mg alloy electrodes do not exhibit any large pores after full delithiation, whereas Li‐metal electrodes show large pores at the interface; in addition, due to the absence of void formation (that could be mitigated by mechanical deformation) in the former, a negligible external‐pressure effect is observed in such systems. Kasemchainan et al. have investigated void formation at the interface, along with the consequent short‐circuit formation.^[^ ^65^ ^]^ In situ X‐ray computed tomography has been used to directly observe the void formation during Li stripping and increase in the number and length of voids along an Li/Li_6_PS_5_Cl interface during cycling (Figure 7g). The number of white pixels (representing voids in the image) at the Li metal/solid electrolyte interface gradually increases with cycling, indicating void accumulation at the interface. Increasing void accumulation on cycling increases the local plating current density, eventually causing dendrite formation through the electrolyte, finally resulting in cell failure. Therefore, for a stable Li‐metal anode/solid electrolyte interface, it is vital to maintain the critical current density for Li stripping and plating. These observations clearly indicate that the morphological instability at the interface during battery operation is a vital factor to ensure long‐term battery performance.

**Figure 8:**
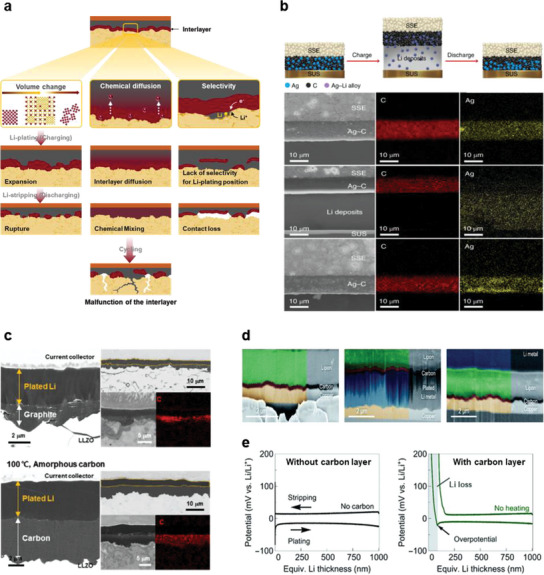
a) Schematic illustration of the degradation mechanism at the interface of a solid electrolyte and Li‐metal anode with interlayers during repetitive Li plating and stripping. The failure of a cell with bare Li metal can be initially retarded using the conventional interlayers. However, the (electro)chemical/physical instability of the interlayer (attributed to a large volume change, chemical mixing/dissolution of the interlayer into Li metal, and the lack of lithium‐plating‐position control) eventually causes cell failure, as shown in the diagrams. Reproduced with permission.^[^ ^81^ ^]^ Copyright 2022, American Chemical Society. b) (Top) Schematic illustration of Li plating–stripping on a current collector with an Ag–C nanocomposite layer during charging and discharging. (Bottom) Cross‐sectional SEM and corresponding EDS images for the Ag–C nanocomposite layer in the pristine condition, and after charging and discharging at 0.1 C, at 60 °C. Reproduced with permission.^[^ ^79^ ^]^ Copyright 2020, Springer Nature. c) Cross‐sectional SEM and EDS images of LLZO solid electrolyte/anode interfaces after the electrodeposition of Li on the (left) graphite layer and (right) amorphous carbon layer at 100 °C. Reproduced with permission.^[^ ^81^ ^]^ Copyright 2022, American Chemical Society. d,e) Li plating and stripping on a current collector through an LiPON solid electrolyte with a carbon layer. Cross‐sectional SEM and EDS images of the carbon interlayer at the current collector/LiPON solid electrolyte interface in the pristine condition, after plating an Li layer (with a thickness of 1 µm), and after stripping the plated Li at a current density of 0.2 mA cm^−2^. Voltage profiles during Li plating and stripping on a Cu foil without and with carbon films. Reproduced with permission.^[^ ^82^ ^]^ Copyright 2022, The Royal Society of Chemistry (e).

However, it is difficult to sustain intimate contact between the solid electrolyte and bare Li‐metal anode, owing to the diffusion limitation of Li metal and imperfections on the solid–electrolyte surface, which cause non‐uniform current distribution and void formation. Therefore, an anode structure that can enhance Li‐atom transport is required to maintain intimate contact during repetitive Li plating/stripping. Li‐metal alloys, such as Li–In and Li–Mg, are unsuitable as anodes due to their unacceptable anode‐operation voltages and battery‐cell energy densities. Considering the practical cell‐manufacturing process, applying sufficient external pressure to induce the physical deformation of Li metal is also not a viable strategy. Although introducing a thin interlayer could be a feasible anode‐modification strategy, most interlayers presented in the previous section are inappropriate owing to their limitations (which depend on their working mechanisms). As schematically shown in **Figure** **8**a, interlayer materials that undergo reactions with Li metal, such as alloying^[^ ^14^, ^27^, ^30^, ^31^, ^71^, ^72^, ^73^, ^74^ ^]^ or conversion,^[^ ^24^, ^35^, ^48^, ^75^, ^76^, ^77^ ^]^ exhibit significant volume changes during these reactions. As reported by Kim et al., such metal interlayers undergo morphological changes during Li plating (alloying) and stripping (dealloying) and do not recover their original shapes.^[^ ^66^ ^]^ Moreover, chemical diffusion between the metal species that form the alloy phase is thermodynamically favored, and the reaction continues until thermodynamic equilibrium is reached;^[^ ^78^ ^]^ this indicates that the alloying metal interlayers are gradually diluted in the Li metal matrix, eventually causing interlayer‐loss. Thus, to maintain interfacial stability during prolonged battery cycling, it is critical to select an appropriate interlayer for application, considering its morphological and chemical changes/sustainability during battery operation.

Carbon‐based interlayers exhibit high potential as interlayers that ensure a stable anode/electrolyte interface.^[^ ^79^, ^80^, ^81^ ^]^ According to Lee et al. (as shown in Figure 8b), a thin Ag–C composite anode without a Li reservoir, which works as an interlayer for uniform Li plating, enables the construction of high‐performance all‐solid‐state batteries that operate at practicable current densities, overcoming the interfacial‐instability issue.^[^ ^79^ ^]^ These batteries exhibit a long cycle‐life (1000 times greater than that of ASSBs) with a high energy density (>900 WhL^−1^). As the chemical and physical durability of interlayers are vital for stable battery operation, the results of this study indicate that the Ag–C composite layer maintains its structure during long‐term battery operation, preventing Li penetration into the solid electrolyte. It has been verified that dense Li metal is uniformly precipitated on the current collector (through the Ag–C layer) during the charging process, which completely disappears in the subsequent discharging process. Despite a redistribution of the Ag component within the Ag–C layer, the layer retains its morphology after long‐term cycling. According to the proposed working‐mechanism of the Ag–C layer, Ag nanoparticles lower the Li metal nucleation energy and provide precipitation sites during the charging process, thereby inducing uniform and dense Li‐metal plating. Suzuki et al. have verified the efficacy of Ag components in Ag–C layer by comparing the performances of batteries with other metal‐nanoparticle components, such as Zn, Al, Sn, and Ni, in metal–C composite anodes.^[^ ^80^ ^]^ Only the Ag–C composite layer shows effective short‐circuit suppression, with a better rate capability and capacity retention than the other metal composite layers. Although both these studies have investigated the role and mechanism of the metal–C composite interlayer and reported excellent battery performance, they have focused on the metal component (rather than the carbon component); thus, the role of the carbon component and its mechanism of action remains unclear. Subsequently, Kim et al. have systematically investigated the activity of the carbon component during the Li plating and stripping processes;^[^ ^81^ ^]^ it guides Li‐metal plating and stripping during battery‐cell operation. Theoretical calculations indicate that Li plating on the current collector (through the carbon layer) is energetically more favorable than Li plating between the carbon layer and solid electrolyte. DFT calculations of interfacial‐energy changes with the Li‐plating position indicate preferential Li plating toward the current collector (i.e., Li plating between the carbon layer and solid electrolyte), rather than Li plating between the current collector and solid electrolyte. The interfacial energy is lowered to −1.839 J m^−2^ for Li plating onto the current collector, whereas Li plating between the current collector and solid electrolyte involves an interfacial energy of −1.362 J m^−2^. Figure 8c shows the cross‐sectional SEM and EDS images of LLZO solid electrolyte/anode interfaces after the electrodeposition of Li through a graphite or amorphous carbon layer at 100 °C at various magnifications. This cross‐sectional analysis validates the preferential plating of Li between the carbon interlayer and current collector, regardless of the type of carbon, in agreement with theoretical predictions (using DFT calculations). Li plating on the current collector through a carbon interlayer has been verified in an anode‐free SSB system using a lithium phosphorus oxynitride (LiPON) electrolyte. As reported by Futscher et al., Li metal is uniformly plated on the current collector after passing through a 150‐nm‐thick amorphous carbon layer, deposited by direct‐current magnetron sputtering as an intermediate layer between the Cu current collector and LiPON solid electrolyte, as shown in Figure 8d.^[^ ^82^ ^]^ Other than verifying the role of the carbon interlayer in guiding the Li‐plating location, similar to previous publications,^[^ ^79^, ^81^ ^]^ this study confirms that the carbon layer functions as a seed layer, ensuring uniform Li plating by lowering the nucleation energy barrier (which appears as an overpotential at the beginning of Li plating) (Figure 8e). This study reports an overpotential of ≈100 mV for Li nucleation on bare Cu foil, which reduces to ≈10 mV after the application of carbon interlayers. Owing to carbon interlayers, anode‐free thin‐film solid‐state batteries composed of Cu/C/LiPON/LCO/Au show excellent electrochemical performance, with a high rate capability and cycle retention compared to cells without carbon interlayers.

**Figure 9:**
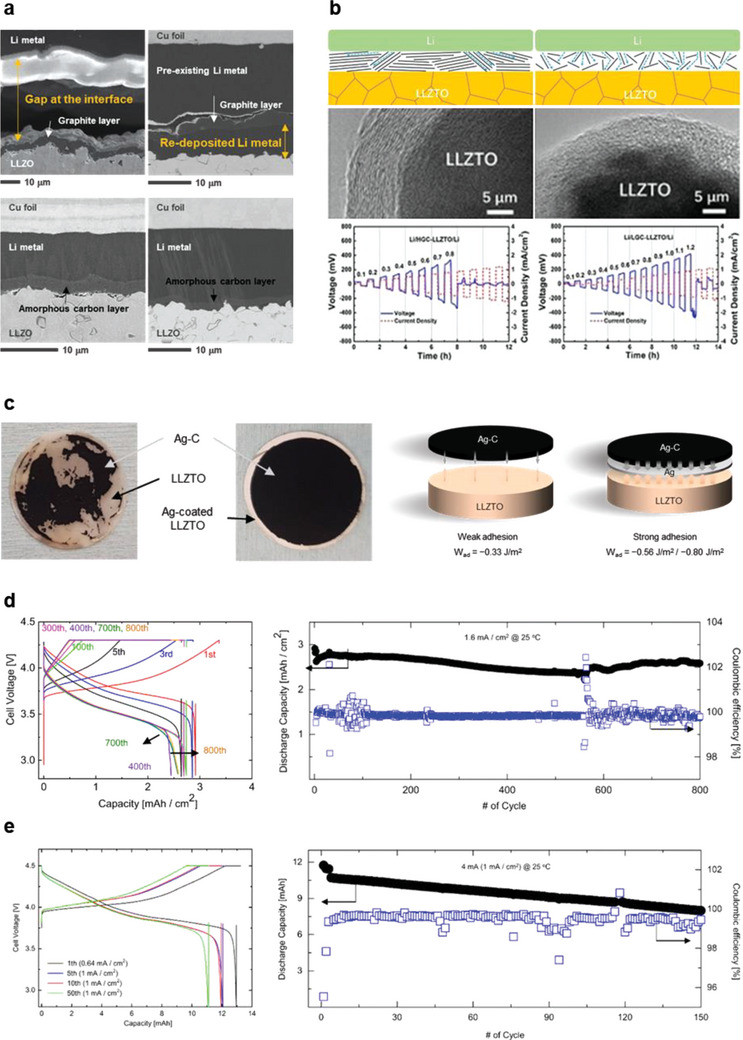
a) Cross‐sectional SEM images of an LLZO solid electrolyte/Li‐metal anode interface with the interlayers after Li stripping and subsequent Li plating. Reproduced with permission.^[^ ^81^ ^]^ Copyright 2022, American Chemical Society. b) Diagrams of HGC and LGC lithiation pathways with the corresponding TEM images of the coated LLZTO electrolyte and CCD test results of the symmetric cells with HGC and LGC modifications. The current density has been increased from 0.1 to 1.5 mA cm^−2^. Reproduced with permission.^[^ ^86^ ^]^ Copyright 2020, Wiley‐VCH. c) (Left) Optical image of the LLZTO electrolyte after the transfer of the Ag–C interlayer and (Right) Schematic models of LLZTO/interlayer interface without and with the Ag layer, indicating the adhesion enhancement. Reproduced with permission.^[^ ^87^ ^]^ Copyright 2023, Springer Nature. d,e) Galvanostatic voltage profiles and cycling performance of a Li|Ag‐C/Ag/LLZTO/IL|NCM333 (3.2 mAh cm^−2^) single‐layer pouch cell (d) and Li|Ag–C/Ag/LLZTO/IL|LCO cell with a capacity of 12 mAh, with an Ag/Ag–C anode interlayer at 25 °C, without external pressure. Reproduced with permission.^[^ ^87^ ^]^ Copyright 2023, Springer Nature (e).

However, the electrochemical performance differs with the type of carbon. Cells with a graphite interlayer show lower capacities (3.3 mAh cm^−2^ at 1.6 mA cm^−2^) than those with an amorphous carbon interlayer (4.3 mAh cm^−2^ at 1.6 mA cm^−2^) and fail at relatively lower current densities (the former show short‐circuit formation at 2.0 mA cm^−2^, whereas the latter operate without short‐circuit formation at 2.5 mA cm^−2^).^[^ ^81^ ^]^ Li stripping behavior depends on the type of carbon and significantly affects the subsequent Li plating. **Figure** **9**a shows the cross‐sectional SEM images of the LLZO solid electrolyte/Li‐metal anode interface with interlayers, after Li stripping. A large gap is observed at the graphite interlayer/Li metal interface throughout the sample, while the amorphous carbon layer maintains intimate physical contact with the Li metal, indicating that the interlayer plays a critical role in Li stripping and plating. The Li‐transport pathway, which depends on the crystallographic characteristics of the material, possibly affects the current distribution, and consequently, the contact at the interface. As graphite is constructed by stacking graphene basal planes, 2D Li transport is expected through it,^[^ ^83^ ^]^ which leads to a localized current distribution and nonuniform Li stripping. In contrast, amorphous carbon is expected to provide isotropic and 3D Li transport pathways because of its highly disordered structure;^[^ ^84^, ^85^ ^]^ this induces a uniform current distribution that could mitigate void formation. With the formation of voids in the Li metal in contact with the interlayer, Li plating becomes increasingly inhomogeneous and causes Li dendrite penetration (regardless of the interlayer maintaining contact with the solid electrolyte). This is consistent with a report by Feng et al.^[^ ^86^ ^]^ In this study, a few nanometers of carbon modification layers have been deposited at the Li metal/LLZTO electrolyte interface by thermal‐decomposition vapor deposition (TVD); symmetric cells with these carbon layers show higher critical current density values (0.8 mA cm^−2^ or 1.2 mA cm^−2^) than those without them (0.3 mA cm^−2^). Cells with lower graphitized carbon (LGC) show better performance (1.2 mA cm^−2^) than cells with a highly graphitized carbon (HGC) layer (0.8 mA cm^−2^), indicating a relatively more rapid transport of Li^+^ through LGC (than HGC) due to the presence of more pathways in LGC owing to its disordered structure (Figure 9b). Thus, along with the number of Li pathways, a uniform distribution of Li pathways is important for facile Li^+^ transport. Summarizing, rapid and uniform Li distribution through the interlayer during battery operation (influenced by the Li‐transport kinetics) and a chemically and physically stable interface are vital for reliable electrochemical battery performance. Carbon‐based interlayers could guide anode‐design strategies for the practical implementation of SSBs.

Notably, in addition to the Li metal/interlayer interface, the interlayer/solid electrolyte interface should be stable for good battery performance. If the interlayer loses contact with the solid electrolyte, the current distribution at the interface becomes inhomogeneous, adversely affecting the interfacial stability. Recently, Kim et al. have reported the long‐term cycling of a high‐energy‐density (≈690 Wh L^−1^) solid‐state Li‐metal battery with a thin LLZTO electrolyte using a hierarchical‐structure anode interlayer.^[^ ^87^ ^]^ In this study, an additional 200‐nm‐thick Ag layer has been deposited on the LLZTO solid electrolyte to improve the interfacial stability between the solid electrolyte and Ag–C composite layer. As shown in Figure 9c, an additional Ag layer facilitates the transfer of the Ag–C layer onto the LLZTO solid electrolyte, indicating enhanced adhesion. This enhanced adhesion is confirmed by DFT calculations; the calculated work of adhesion between LLZTO and Ag is −0.80 J m^−2^, whereas that between Ag and the Ag–C composite is −0.56 J m^−2^. Apart from this, ab initio molecular dynamics (AIMD) simulations show that the LLZTO/Li_9_Ag_4_ interface remains intact for at least 40 ps, indicating the chemical stability of the interface between LLZTO and the Ag/AgC interlayer. Thus, the stability of the interface between the Ag–C layer and LLZTO electrolyte is expected to benefit from the Ag layer, which provides additional physical and chemical stability. In addition, the Ag layer possibly enhances the Li‐diffusion rate in the anode; the papers published by Lee et al. and Kim et al. do not state this information.^[^ ^79^, ^87^ ^]^ According to Krauskopf, Li diffusion is faster in Li alloy materials compared to that in pure Li metal.^[^ ^70^ ^]^ Li–Ag has also been reported to induce rapid Li diffusion.^[^ ^33^, ^66^ ^]^ As reported by Jin et al., cells with a Li–Ag alloy exhibit a better battery performance than those with bare‐Li cells.^[^ ^33^ ^]^ This can be ascribed to a higher Li diffusivity (≈10^−8^ cm^2^ s^−1^) in Li–Ag alloys compared to that in bulk‐Li metal (≈10^−11^ cm^2^ s^−1^). Thus, according to the results of both Lee et al. and Kim et al., the kinetics of Li transport at the interface and in the anode can be improved by introducing an Ag component. Eventually, Li|Ag‐C/Ag/LLZTO/IL|NCM333 cells with this hierarchically structured interlayer operate with an initial areal discharge capacity of ≈3 mAh cm^−2^ and can be stably cycled (for over 800 cycles) at 1.6 mA cm^−2^ and 25 °C (Figure 9d). In addition, as shown in Figure 9e, this study reports a cell with a capacity of 12 mAh under more practical conditions; the cell uses a large‐area thin solid electrolyte and is operated under a current of 4 mA without external pressure. The sustained stability of the interfaces between the Li‐metal anode and LLZTO solid electrolyte during long‐term cycling can be attributed to the combination of the suggested roles of the carbon‐based interlayer and the Ag layer; the Ag–C layer guides Li plating to preferentially take place on the current collector, acts as a partially porous anode (refer to the additional discussion in Section 2.3), and enables fast and uniform Li distribution. Ultimately, the Ag layer contributes to sustain the stable interface between the Ag–C layer and LLZTO electrolyte during repetitive Li plating and stripping.

Although optimal interlayer design, considering the degradation mechanisms of the interlayer upon Li plating and stripping, shows high potential as a beneficial anode component, several issues remain unresolved. First, the volume change of Li‐metal anodes during plating and stripping is a critical obstacle hindering the implementation of solid‐state Li metal batteries in real‐world applications. Second, inherent defects in the solid electrolytes could render the anode interface non‐uniform. Therefore, these defects could potentially lead to the redox reaction preferentially occurring at the interface,^[^ ^88^, ^89^ ^]^ which would eventually result in the formation of dendrites.^[^ ^90^ ^]^ Even if the interlayers discussed in this section were used to stabilize the interface, this irregularity at the surface of solid electrolytes could undermine the efficacy of the interlayers. Thus, it is vital to develop an anode structure that can release or accommodate the volume expansion as well as mitigate the issues arising from the defects in the surface of the electrolyte.

### 2.3 Strategies to Minimize Anodic Stress Accumulation and Volume Change

To overcome the limitations discussed in the previous section, it is critical to design and develop an advanced interlayer or anode architecture. Anodes with a 3D porous architecture could alleviate the local stress evolution and subsequent volumetric changes during cycling. Anodes with 3D structures provide an initial free volume that can accommodate Li deposits during charging. This anode architecture shows several advantages over dense anodes. First, it mitigates the local stress evolution during Li plating. Due to the low melting temperature of Li metal, the plated Li can be transported away from the solid–electrolyte surface via creep.^[^ ^91^, ^92^, ^93^ ^]^ Therefore, the deposition of Li metal during charging generates minimal stress at the solid electrolyte/Li deposit interface, which effectively prevents Li metal penetration through the solid electrolyte. In contrast, in the absence of such a stress‐relaxation pathway, an enormous local stress is generated by Li plating, which is capable of fracturing CNTs.^[^ ^94^ ^]^ In such cases, fragile solid electrolytes, such as LLZO, are easily fractured, resulting in short circuiting. In addition, 3D porous anodes prevent anodic volume changes. As it is possible to calculate the volume occupied by Li deposits, the careful design of the 3D‐anode structure (considering the porosity and thickness) could enable a zero anodic volume‐change during cycling. Thus, 3D porous anodes can be used for the efficient construction of safe and strain‐free solid‐state batteries.

**Figure 10:**
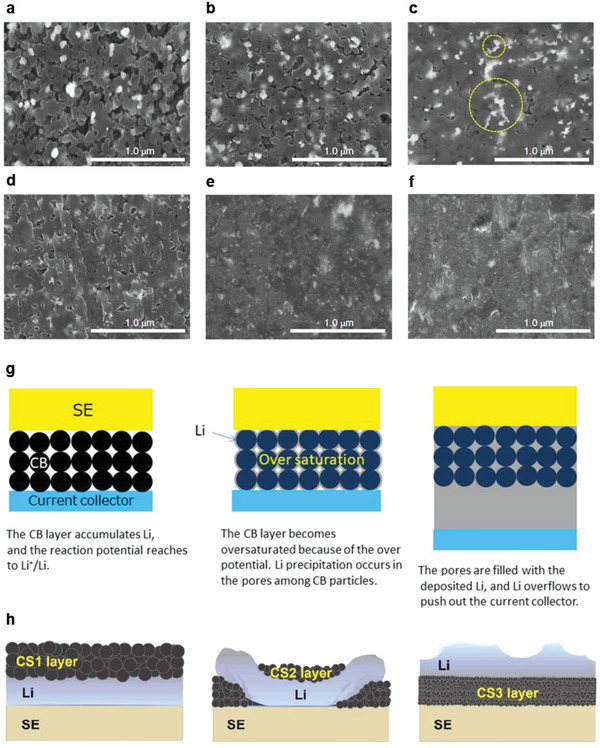
Cross‐sectional SEM images of the middle part of an Ag–C nanocomposite layer at a) the pristine condition, b) 3.5 V, c) 3.55 V, d) 3.6 V, e) 4.0 V, and f) 4.25 V. Reproduced with permission.^[^ ^79^ ^]^ Copyright 2020, Springer Nature. g) Schematic figures of the evolution of all‐solid‐state batteries with carbon black‐based anodes during charging. Reproduced with permission.^[^ ^80^ ^]^ Copyright 2021, Wiley‐VCH. h) Schematic illustrations of Li in carbon‐sphere composite anodes deposited to a capacity of 3 mAh cm^−2^. CS1, CS2, and CS3 layers indicate carbon‐sphere composites with diameters of 1 µm, 350 nm, and 75 nm, respectively. Reproduced with permission.^[^ ^95^ ^]^ Copyright 2022, The Royal Society of Chemistry.

Carbon‐based interlayers, which have been extensively reviewed in the previous section, can be classified as 3D porous anodes.^[^ ^79^, ^80^, ^81^, ^87^, ^95^, ^96^ ^]^ The Ag–C composites reported by Lee et al.^[^ ^79^ ^]^ show apparent porosity, even after a warm isostatic press, indicating that the composites provide some space for Li‐metal plating. As shown in **Figure** **10**a–f, they exhibit a reduction in porosity during charging, possibly due to C lithiation; this indicates that the plated Li metal fills the pre‐existing empty space. Therefore, during the first stage of charging, the cell benefits from the 3D porous architecture of the Ag–C composite. However, the initial pore volume of the Ag–C composite is not sufficiently large to completely accommodate the volume generated by Li plating on the cathode composite (6.8 mAh cm^−2^). Thus, after the initial pore space is fully filled by Li metal, further charging generates a new volume of Li deposits, leading to local mechanical stress and a volume change. A study reports 25‐µm‐thick Li deposits after the first charge,^[^ ^79^ ^]^ indicating that the cell‐anode should undergo a volume change of ≈25 µm in each charge/discharge cycle. Notably, although the Ag–C composite used by Lee et al. partially acts as a 3D porous anode, their primary design strategy was not to utilize the anode‐structure porosity. Suzuki et al. have utilized carbon layers, including pores, as anodes; the corresponding cells exhibit an excellent capacity retention of ≈87% at the 150th cycle.^[^ ^80^ ^]^ According to their analysis, Li metal first precipitates at the pores between the carbon‐black particles, and then, overflows toward the current collector (Figure 10g). Interestingly, graphite‐based anodes do not suppress short circuits, possibly due to the large particle‐size of graphite (≈15 µm) compared to that of carbon black.

Recently, Park et al.^[^ ^97^ ^]^ have systematically investigated the effect of pore size on a Ni 3D porous anode. Under a controlled charge capacity and current density (2 mAh cm^−2^ and 0.5 mA cm^−2^, respectively), Li‐deposition behavior varies with the pore size of the anode at elevated temperatures (45 °C). Li metal is not infiltrated into a porous host structure with a large pore size of 3.58 µm; however, more than half of the plated Li advances toward porous anodes with small pores (314 nm), occupying the pore space between Ni particles. This indicates that Li metal transport kinetics depends significantly on the porous morphology of the host structure. The authors of this study have interpreted this distinct behavior using different Li‐creep mechanisms. When the pore size is large, Li deformation predominantly occurs by a displacive mechanism, whereas small pores induce the diffusional Coble creep along the pore surface.^[^ ^98^, ^99^ ^]^ According to Park et al., plated Li does not grow inside anodes with large pores because displacive deformation requires a much higher stress than the diffusional Coble creep mechanism. Instead of filling the pores, Li continuously accumulates at the solid electrolyte/anode interface, creating local mechanical stress, which eventually separates the solid electrolyte and anode. Another publication reports an identical trend with carbon‐based composite anodes at 60 °C.^[^ ^95^ ^]^ As shown in Figure 10h, composite anodes composed of small‐sized carbon spheres (≈75 nm) cause the facile deformation of Li metal; therefore, Li advances toward the composite‐layer pores after charging. Similar to the Ni‐anode experiment, Li does not fill the pores between large‐sized carbon spheres (≈1 µm); therefore, Li plating occurs at the solid electrolyte/composite layer interface. Therefore, the transport behavior of Li metal is independent of the anode material, emphasizing the importance of the microstructural design of 3D porous anodes. The strain rate of Li deformation by Coble creep is correlated with the surface diffusivity of Li metal; therefore, the operational temperature is a critical factor influencing Li‐transport kinetics. For example, as reported by Park et al., all Li deposits do not penetrate composite anodes with small‐sized carbon spheres (≈75 nm) at 30 °C, whereas they do so at 60 °C.^[^ ^95^ ^]^ Similarly, as reported by Kim et al., plated Li‐metal remains at the solid electrolyte/graphite interlayer interface at 25 °C, separating them, whereas all the Li metal penetrates into the interlayer and is plated between the interlayer and current collector at 100 °C.^[^ ^81^ ^]^ These studies highlight the importance of considering the transport kinetics of Li metal in the design of 3D porous architectures. Although Li is a soft metal, which shows a high homologous temperature and provides empty space for stress‐free deformation, the rigorous design of 3D porous structures that could maximize Li deformation kinetics is vital for the fabrication of a stable and efficient Li‐anode.

**Figure 11:**
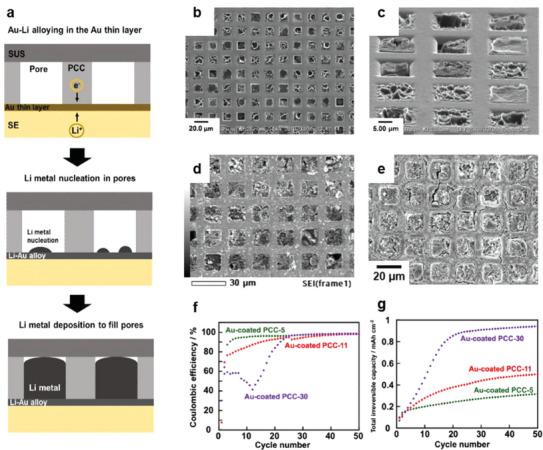
a) Schematic illustration of Li plating on a solid electrolyte with a porous current collector. b,c) SEM images of PCC‐11 after Li plating at 0.064 mA cm^−2^ for 15 h. SEM images of d) the front side of PCC‐11 and e) the solid electrolyte surface after the 15th (1.5 mA cm^−2^) plating/stripping of the PCC‐11 | PLSLI | Li–In cell. f) Comparison of coulombic efficiencies of cells with Au‐coated PCC‐30, Au‐coated PCC‐11, and Au‐coated PCC‐5 for 50 cycles. g) Total irreversible capacities of cells with Au‐coated PCC‐30, Au‐coated PCC‐11, and Au‐coated PCC‐5. PCC‐30, PCC‐11, and PCC‐5 indicate porous current collectors with pore‐aperture sizes of 30, 11, and 5 µm, respectively. a‐g) Reproduced with permission.^[^ ^100^ ^]^ Copyright 2021, American Chemical Society.

In addition to constructing 3D structures with traditional anode materials such as carbon, studies have attempted to fabricate 3D porous current collectors.^[^ ^100^, ^101^, ^102^, ^103^ ^]^ As Li‐metal plating and stripping occur due to electrochemical reactions at the anode, 3D host materials are not required to exhibit Li‐ion conductivity. Therefore, simple electronic conductors, such as current collectors, can be used as host materials for 3D frameworks. Shinzo et al. have utilized a commercially available Ni electroforming sieve with well‐ordered square pores (**Figure** **11**a) as the porous current collector framework.^[^ ^100^ ^]^ Three porous current collectors with different aperture sizes and porosities have been fabricated using 11 µm‐thick PCC‐11, with a back‐side aperture size of 11 µm and porosity of 46%; Li metal fills the pores at a current density of 0.064 mA cm^−2^ for an areal capacity of 0.96 mAh cm^−2^, which corresponds to 90% pore‐filling, as shown in Figure 11b,c. Modulating the solid‐electrolyte composition from Li_3_PS_4_ to a Li_3_PS_4_‐LiI composite reduces SEI formation, and coating the PCC‐11 surface with Au facilitates the diffusion of the deposited Li, enabling the system to exhibit a high current density of over 6.0 mA cm^−2^. Thus, a well‐aligned 3D porous structure hinders electrolyte fracture and subsequent short‐circuit formation. The Li‐deposition behavior using porous current collectors with randomly distributed pores has been compared to those with a well‐aligned pore distribution;^[^ ^102^ ^]^ although a rigorous comparison is difficult because of the different thicknesses and pore sizes, it has been confirmed that Li completely fills the randomly distributed pores at a relatively low current density (0.064 mA cm^−2^). Ma et al. have fabricated a porous Cu current collector with random 3D surface structures using electrodeposition;^[^ ^103^ ^]^ it contains nanometer‐sized clusters which facilitate rapid Li deformation kinetics. In combination with Li_6_PS_5_Cl, the porous Cu current collectors deliver a larger capacity than planar Cu current collectors, after 20 cycles. Although these studies report metal‐based 3D frameworks, as the electrochemical reaction occurs at the interface of the solid electrolyte and 3D‐framework surface, these 3D structures can also be constructed using insulating materials coated with an electron‐conducting material. Shinzo et al. have spatter‐coated Au or Ni onto the sidewalls of the pores in commercially available polycarbonate membrane filters to create electrical connections.^[^ ^101^ ^]^ Similar to the aforementioned studies, Li metal fills the pores of the metal‐coated polycarbonate membrane filters. Thus, 3D porous structures are useful for Li plating. As reported by multiple studies, Li metal fills the pores of such structures, unless kinetically prohibited, and 3D anodes generate a high critical current density. However, such systems generate a low coulombic efficiency due to irreversible Li stripping. For example, as reported by Shinzo et al., the irreversible capacity is constantly accumulated during extended charge/discharge cycles using PCC‐11.^[^ ^100^ ^]^ Moreover, SEM images clearly indicate that a considerable amount of Li remains in the pores of PCC‐11 after 15 cycles (Figure 11d,e); thus, incomplete Li stripping is a major bottleneck preventing high capacity retention. Li vacancy transport during Li stripping is slower than the typical discharge current density, causing pore formation at the solid electrolyte/Li metal interface.^[^ ^3^ ^]^ Therefore, a stack pressure is applied with planar‐architecture anodes to aid Li transport and reduce pore formation.^[^ ^61^, ^104^, ^105^ ^]^ However, this strategy is impractical for 3D porous anodes. A uniaxial mechanical load is normally applied to the cell to apply pressure; however, the Li metal inside the 3D porous anode is not influenced by the external load. Assuming that Li fully fills the pores of the 3D anodes after charging, the Li metal body is physically connected to the current collector and solid electrolyte; at this stage, stack pressure can be applied to the Li metal. However, Li starts to disconnect from the current collector when Li stripping occurs during discharge because the rigid 3D framework prevents cell shrinkage. In this case, pressure cannot be applied to the Li metal, resulting in incomplete Li stripping and a low coulombic efficiency (Figure 11f,g). Hence, alternative approaches, such as pre‐lithiation, must be devised to mitigate the irreversibility and reduce the pore size, to encourage the contribution of the diffusional Coble creep mechanism.

3D frameworks can also be constructed using solid‐state electrolytes.^[^ ^24^, ^106^, ^107^, ^108^, ^109^, ^110^ ^]^ For instance, etching the surface of a solid electrolyte with acids has been used to form porous layers at the anode interface.^[^ ^111^, ^112^ ^]^ Ruan et al. introduced a 3D cross‐linking porous layer by treating the surface of LLZTO with an acidic solution containing HCl and HF.^[^ ^111^ ^]^ Similarly, Chen et al. constructed lithiophilic 3D layers on garnet by etching the LLZTO surface with a 40% HF solution.^[^ ^112^ ^]^ Although etching with an acid could be utilized to form 3D electrolyte structures, this approach has a clear limitation because the effect of surface etching is generally restrained to very thin (≈2 µm) layers on the surface.

**Figure 12:**
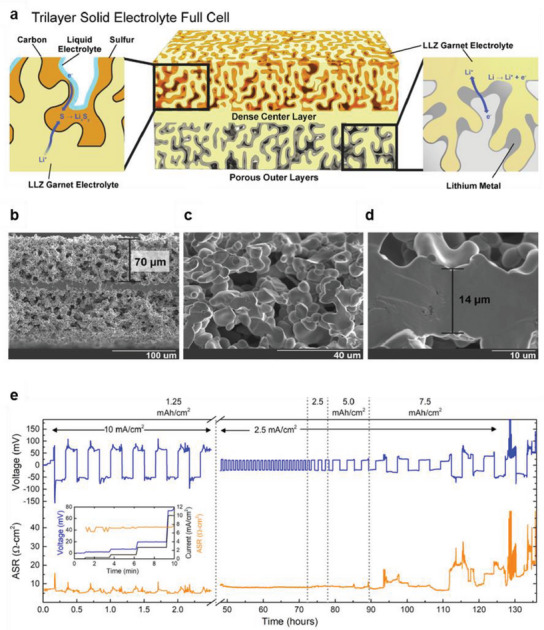
a) Diagram of a trilayer Li garnet cell, where the trilayer is filled with active materials to create a Li‐metal battery. b) SEM cross‐section of the porous‐dense‐porous trilayer after binder burnout and sintering. Magnified SEM of the c) porous layer with high porosity and interconnectivity and d) thin, dense blocking layer of a sintered trilayer cell. e) Cell voltage response (blue) and ASR (orange) during galvanostatic cycling of the trilayer Li symmetric cell at 10 and 2.5 mA cm^−2^. a‐e) Reproduced with permission.^[^ ^106^ ^]^ Copyright 2018, Elsevier.

In this regard, attempts have been made to fabricate thick (>10 µm) layers of porous solid electrolytes.^[^ ^113^, ^114^ ^]^ Bao et al. successfully prepared a self‐standing LLZTO ceramic skeleton with a thickness of 10−15 µm using a tape‐casting method.^[^ ^113^ ^]^ Further, Okur et al. fabricated a porous LLZO scaffold with intermediate‐stage sintering, without the use of any pore formers.^[^ ^114^ ^]^ Although these studies demonstrated that porous solid electrolytes can be produced, the porosity of the solid electrolyte would have to be much higher to fully utilize the benefits of 3D porous structures. Moreover, because solid electrolytes are theoretically not electron conductors, they should be coated with conductive agents to facilitate electrochemical reactions at the solid–electrolyte surface and reversibly store Li metal during charge and discharge. This strategy has mostly been implemented using garnet‐type solid electrolytes. For example, Xu et al. have fabricated porous‐dense‐porous trilayer garnets using cross‐linked polymethyl methacrylate (PMMA) spheres as porogens,^[^ ^107^ ^]^ followed by a CNT‐filling of the pores by the repeated infiltration of CNT ink; this imparts electronic conductivity to the surface of the porous‐garnet framework. Li deposits can be infused into the pores of 3D mixed electron/ion‐conducting frameworks (MCFs) during charging, similar to the previously mentioned 3D porous anodes or current collectors. However, a striking difference is observed between the two systems. In 3D MCFs, the entire surface of the 3D framework is electrochemically active because both Li ions and electrons can be provided at the surface; Li ions are supplied through the framework (solid electrolyte) and electrons are transported from the current collector to the surface‐coating layer. Contrarily, 3D electron‐conducting frameworks exhibit a limited active surface area, which is the contact area between the solid electrolyte and porous framework. Therefore, 3D MCFs efficiently lower the effective current density, which is the applied current density normalized by the active surface area. According to Hitz et al., symmetric cells using a porous‐dense‐porous trilayer garnet as a 3D MCF can be cycled at a current density of 10 mA cm^−2^ with a capacity of 1.67 mAh cm^−2^ (**Figure** **12**).^[^ ^106^ ^]^ As the surface area of trilayer MCF is 40 times higher than that of planar garnet, it can exhibit low effective current densities (as low as 0.25 mA cm^−2^) with high applied current densities (as high as 10 mA cm^−2^). This enables facile Li stripping and plating, without an effective external pressure. In addition, this study uses the partial filling of pores by Li metal, instead of conductive agents, to provide an initial electron‐conducting pathway. As Li exhibits electronic conductivity, regardless of the surface coating of the porous garnet framework, some Li on the 3D‐skeleton surface after discharge is sufficient for facilitating electronic conductivity.

**Figure 13:**
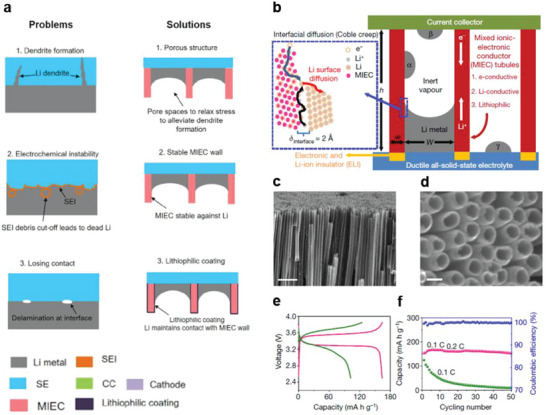
a) Limitations of solid‐state Li‐metal batteries and solutions provided by creep‐enabled 3D solid‐state Li‐metal batteries. Reproduced with permission.^[^ ^98^ ^]^ Copyright 2020, Elsevier. b) Schematic process of creep‐enabled Li deposition/stripping in an MIEC tubular matrix. c,d) Field emission SEM images of carbonaceous MIEC tubules. Scale bars: 1 µm (c) and 200 nm (d). e) Charge/discharge profiles at 0.1 C and f) the cycling life of all solid‐state Li‐pre‐deposited MIEC | SE | LiFePO_4_ batteries. Magenta and blue colors indicate the use of 3D MIEC tubules as the Li host, while green color indicates the use of 2D carbon‐coated Cu foil as the Li host. b‐f) Reproduced with permission.^[^ ^91^ ^]^ Copyright 2020, Springer Nature.

Several studies have extensively investigated the nature of Li plating/stripping in 3D frameworks, qualitatively confirming the factors affecting the Li plating/stripping behavior. Recently, a systematic understanding of the working principle of 3D porous architectures has been used for a quantitative analysis of its elements.^[^ ^91^, ^98^, ^115^ ^]^ In a series of studies, Li et al. have analyzed the limitations of Li‐metal anodes and proposed the utilization of 3D porous anodes. According to their analysis, an ideal anode should rapidly relieve the mechanical stress originating from Li plating and maintain electrochemical stability at the interface and solid electrolyte/Li metal interfacial‐contact during charging and discharging (**Figure** **13**a).^[^ ^98^ ^]^ Based on these requirements, they have proposed a design strategy to construct stable 3D hosts that enable rapid stress relaxation. First, the material for the 3D anode or framework should be mixed with an ion‐electron conductor (MIEC) to provide sites for Li plating/stripping and electron‐conducting pathways, when insulating byproducts are present at the interface.^[^ ^115^ ^]^ Second, the MIEC should be electrochemically stable against Li ions; lithiated carbon or Li alloys, such as Li_22_Si_5_ and Li_9_Al_4_, meet this criterion. Third, an ion‐electron insulator (IEI) should be incorporated at the solid electrolyte/MIEC contact‐area. The IEI will mechanically bind the solid electrolyte and MIEC, constantly maintaining contact by suppressing Li plating/stripping, and will prohibit stress generation at the contact area. If the IEI is in direct contact with the solid electrolyte, Li plating occurs at the interface; this generates immense local stress, making the interface prone to detachment. Finally, careful geometrical design is required to ensure sufficient Li transport that facilitates a high current density (≈3 mA cm^−2^) and shows abundant pore volume, capable of accommodating a high capacity (≈3 mAh cm^−2^). For example, the MIEC fill factor, which approximately corresponds to the ratio of the MIEC width to pore width, should be at least 0.1 for LiC_6_.^[^ ^91^ ^]^ In addition, the anodes should be thicker than 20 µm in a tubular‐matrix geometry. Based on an empirical formula for monatomic metals,^[^ ^116^ ^]^ Chen et al. have postulated that the surface diffusion coefficient of Li is large (≈7 × 10^−7^ cm^2^ s^−1^) at room temperature;^[^ ^91^ ^]^ therefore, Li transport predominantly occurs via interfacial MIEC channels. This indicates that the kinetic issues during Li plating and stripping can be mitigated by enlarging the MIEC‐wall surface. Consequently, Chen et al. have utilized carbon tubules with an inner diameter of ≈100 nm and wall width of ≈20 nm as a 3D host structure, as shown in Figure 13b–d.^[^ ^91^ ^]^ In situ TEM observations of a single carbon tubule coated with lithiophilic ZnO*_x_* indicate that Li can be reversibly plated and stripped along the carbon tubule for more than 6 µm. In addition, void plugs are occasionally formed between the solid electrolyte and plated Li, which do not prevent Li stripping, indicating a Li‐transport pathway along the MIEC surface. Thus, a half‐cell with a 3D framework shows a lower overpotential (39 mV) and higher coulombic efficiency (97.12%) at a current density of 0.125 mA cm^−2^ than a cell with a carbon‐coated 2D Cu foil (250 mV and 74.34%, respectively). Further, full cells with an LiFePO_4_ cathode can be cycled for more than 50 cycles (without noticeable degradation) at 0.2 C and 55 °C, as shown in Figure 13e,f. Thus, the systematic design of the anode 3D‐architecture can facilitate the practical application of all solid‐state batteries.

## 3 Perspectives and Conclusion

Li–metal batteries with solid electrolytes exhibit high potential for practical applications (considering their energy density). However, the solid electrolyte/Li–metal anode interface significantly hinders their practical realization. In addition to chemical and/or electrochemical side reactions, several physical and mechanical issues occur during cycling, which substantially hamper the cell integrity. This review summarizes several approaches for addressing the physical and mechanical issues occurring at the interface. Although interlayers and surface coatings effectively lower the initial interfacial resistance by enhancing contact during cell assembly, they exhibit low stability after long‐term cycling because of morphological evolution at the interface upon repeated Li plating and stripping. Separating the charge‐transfer region (i.e., the anode interface) from the Li‐storage region using carbon‐based interlayers is a viable approach to stabilize the interface under dynamic conditions. Finally, anodes with a 3D porous architecture have been discussed, which seem to be the most promising option to relieve local stress and minimize cell‐volume changes. The working concept of 3D anodes has been confirmed using carbon‐based scaffolds, metal frameworks, and porous solid electrolytes. However, despite extensive research, several hurdles limit the practical application of 3D anodes. First, an irreversible capacity accumulates in the system due to incomplete Li stripping during discharge.^[^ ^100^ ^]^ This incomplete Li stripping has also been reported in cells with conventional dense anodes; it originates from void formation at the Li metal/solid electrolyte interface due to the sluggish self‐diffusion of Li metal at room temperature.^[^ ^3^, ^6^ ^]^ For conventional dense anodes, the application of external pressure is used to resolve this issue. Li metal is ductile and can be easily deformed by external stress; therefore, void formation can be mitigated. However, this approach is unsuitable for 3D anodes because the rigid frameworks prevent the transfer of the applied pressure to Li deposits inside the framework pores. Therefore, alternative strategies should be devised for the operation of 3D anodes under practical conditions (such as at room temperature and current densities higher than 3 mA cm^−2^, in large‐area cells). Coating the surface of a 3D framework, which enables rapid Li diffusion, is a viable strategy. The morphological design of the 3D structure could also be effective because the surface diffusion of Li is expected to be significantly faster than its bulk diffusion.^[^ ^91^ ^]^ 3D porous electrolytes have the advantage of fast Li stripping due to the large active area but only when the homogeneous stripping is guaranteed.

Another important issue is the integration of 3D porous structures with solid electrolytes, particularly brittle oxide electrolytes. It is challenging to establish tight and uniform contact between two rough solids (the solid electrolyte and 3D host). Therefore, it is critical to develop a method for integrating 3D anodes with solid electrolytes. Utilizing a polymer‐based layer at the interface as a contact mediator has proven to be an effective strategy;^[^ ^91^ ^]^ in‐situ polymerization has also been used.^[^ ^117^ ^]^ As 3D hosts have been widely studied as anodes with liquid electrolytes,^[^ ^118^, ^119^, ^120^, ^121^ ^]^ the development of facile integration strategies with solid electrolytes could significantly facilitate the practical application of future all‐solid‐state batteries.

Finally, the anode interface continues to remain problematic in several ways, despite the introduction of a 3D porous structure as the anode instead of Li metal. The non‐uniformity arising from the inherent defects, grain boundaries, or cracks in the surface of solid electrolytes could lead to an irregular redox reaction during charge and discharge, which could potentially result in the premature degradation of anodes. Complications originating from current constriction could be partially resolved by the introduction of 3D porous anodes owing to their increased electrochemically active area and the lower stress or strain at the interface; however, the problem could also be addressed by improving the solid electrolyte. In this regard, amorphous or glass solid electrolytes could be a promising solution.^[^ ^122^, ^123^ ^]^ As these electrolytes do not have grain boundaries, they can effectively eliminate the possible sources of irregularity originating from these defects. However, most amorphous oxide electrolytes exhibit low ionic conductivities (≈10^−6^ S cm^−1^); hence, the thickness of the amorphous oxide layer should ideally be limited to a few micrometers on the surface.

Thus, although several issues require resolution before the commercialization of solid‐state batteries, continuous efforts from the scientific community can accelerate their practical application. This review provides helpful insights regarding Li–metal solid‐state batteries and could guide their practical application in the future, contributing immensely to the advancement of solid‐state battery technology.

## Conflict of Interest

The authors declare no conflict of interest.
